# Erythroid‐specific inactivation of *Slc12a6/Kcc3* by EpoR promoter‐driven Cre expression reduces K‐Cl cotransport activity in mouse erythrocytes

**DOI:** 10.14814/phy2.15186

**Published:** 2022-03-11

**Authors:** Boris E. Shmukler, Alicia Rivera, Katherine Nishimura, Ann Hsu, Jay G. Wohlgemuth, Jeffrey S. Dlott, L. Michael Snyder, Carlo Brugnara, Seth L. Alper

**Affiliations:** ^1^ Department of Medicine Beth Israel Deaconess Medical Center Boston Massachusetts USA; ^2^ Quest Diagnostics Secaucus New Jersey USA; ^3^ Department of Laboratory Medicine Boston Children’s Hospital Boston Massachusetts USA; ^4^ Department of Pathology Harvard Medical School Boston Massachusetts USA; ^5^ Department of Medicine Harvard Medical School Boston Massachusetts USA

**Keywords:** ion transport, membrane protein, radioisotopic flux, red blood cell, tissue‐specific knockout

## Abstract

Investigation of erythrocytes from spontaneous or engineered germ‐line mutant mice has been instrumental in characterizing the physiological functions of components of the red cell cytoskeleton and membrane. However, the red blood cell expresses some proteins whose germline loss‐of‐function is embryonic‐lethal, perinatal‐lethal, or confers reduced post‐weaning viability.

Promoter regions of erythroid‐specific genes have been used to engineer erythroid‐specific expression of Cre recombinase. Through breeding with mice carrying appropriately spaced insertions of loxP sequences, generation of erythroid‐specific knockouts has been carried out for signaling enzymes, transcription factors, peptide hormones, and single transmembrane span signaling receptors. We report here the use of Cre recombinase expression driven by the erythropoietin receptor (EpoR) promoter to generate *EpoR*‐*Cre*;*Kcc3^f^* ^/^ *^f^* mice, designed to express erythroid‐specific knockout of the KCC3 K‐Cl cotransporter encoded by *Kcc3*/*Slc12A6*. We confirm KCC3 as the predominant K‐Cl cotransporter of adult mouse red cells in mice with better viability than previously exhibited by *Kcc3* ^−/−^ germline knockouts. We demonstrate roughly proportionate preservation of K‐Cl stimulation by hypotonicity, staurosporine, and urea in the context of reduced, but not abrogated, K‐Cl function in *EpoR*‐*Cre*;*Kcc3^f^* ^/^ *^f^* mice. We also report functional evidence suggesting incomplete recombinase‐mediated excision of the *Kcc3* gene in adult erythroid tissues.

## Graphical abstract

Erythroid‐specific expression of Cre recombinase in the presence of a floxed Kcc3 gene yields substantial reduction of red cell K‐Cl cotransport activity. However that reduction is less than that achieved by germ‐line KCC3 knockout.

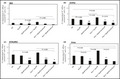

## 1 INTRODUCTION

K‐Cl cotransport is a widely expressed volume regulatory decrease mechanism first described in erythrocytes (RBC) (Dunham et al., 1980; Lauf et al., 1992). K‐Cl cotransport is macroscopically electroneutral and is mediated by the four K‐Cl cotransporters encoded by the SLC12 gene family members (Kahle et al., 2015) SLC12A4/KCC1 (Garneau et al., 2019), SLC12A5/KCC2, SLC12A6/KCC3 (Garneau et al., 2017), and SLC12A7/KCC4 (Marcoux et al., 2017). KCC3 is the dominant K‐Cl cotransporter of normal mouse red blood cells (RBC) (Rust et al., 2007) and in RBC of mouse models of sickle cell disease and β‐thalassemia (Rust et al., 2007; Shmukler et al., 2019, 2020). However, global knockout of *Kcc3* in the mouse partially phenocopies the human KCC3 loss‐of‐function disease, Andermann syndrome, characterized by severe peripheral neuropathy with variable agenesis of the corpus callosum (Boettger et al., 2003; Ding & Delpire, 2014; Howard et al., 2002). *Kcc3* knockout mice also exhibit arterial hypertension and slowly progressive deafness (Rust et al., 2006). In our hands, *Kcc3* ^−/−^ pups survived weaning at 50% of predicted numbers, and only 63% of these survived to 6 weeks of age (Shmukler et al., 2019). Although *Kcc1* ^−/−^; *Kcc3* ^−/−^ mice remained fragile and showed further reduction in erythroid K‐Cl cotransport activity, they paradoxically showed better survival than *Kcc3* ^−/−^ mice. Births of *Kcc3* ^−/−^ and *Kcc1* ^−/−^; *Kcc3* ^−/−^ genotypes were fewer still on the genetic background of the SAD mouse model of sickle disease, chosen for its faithful reproduction of the cellular dehydration phenotype of human sickle red cells (Rust et al., 2006; Shmukler et al., 2019) and its relative ease of breeding and genetic analysis compared to other mouse models of sickle cell disease.

The morbidity and reduced survival of *Kcc3* ^−/−^ mice, in the context of ion transport assay volume requirements best satisfied by blood volumes of adult mice, led us to attempt generation of mice with erythroid‐specific knockout of *Kcc3*. We were encouraged in this effort by previous reports of tissue‐specific, temporally regulated Cre‐induced knockout as well as re‐expression of *Kcc3* (Shekarabi et al., 2012; Flores & Delpire, 2021). Our eventual intention was to study further the role of the KCC3 K‐Cl cotransporter in red cells of the SAD mouse model of sickle cell disease, as well as in other genetic backgrounds allowing the generation of larger numbers of experimental animals that would survive to maturity and provide larger blood volumes for subsequent functional analysis. We were also interested in assessing and, hopefully, confirming the efficacy of the Cre‐EGFP fusion protein under the control of the erythropoietin receptor (*EpoR*) promoter (Heinrich et al., 2004) for erythroid‐specific knockout of erythroid membrane solute transport proteins in adult mice. EPOR‐Cre‐GFP or its codon‐optimized version EPOR‐iCre‐GFP have been previously used for erythroid‐specific genetic inactivation of transcription factors (Dewamitta et al., 2014; Esteghamat et al., 2013; Vassen et al., 2014), signaling enzymes (Jayapal et al., 2016; Liddicoat et al., 2016; Xie et al., 2020), a peptide growth factor (Drogat et al., 2010), and a single‐span transmembrane signaling protein (Wei et al., 2019). Although pan‐hematopoietic knockout of at least three distinct transmembrane proteins has been achieved using VAV1‐Cre‐mediated genomic excision (Cahalan et al., 2015; Rishi et al., 2016; Wang et al., 2020), engineered erythroid‐specific knockout of membrane solute transporters or ion channels by Cre‐mediated recombination under the control of the EpoR promoter has not been reported in mature circulating adult RBC.

## 2 METHODS

### 2.1 Mouse breeding and genotyping

K‐Cl cotransporter‐deficient mice *Kcc1* ^−/−^ and *Kcc1* ^−/−^; *Kcc3* ^+/−^ mice were genotyped as previously described (Rust et al., 2007). *EpoR*‐*Cre*; *Kcc3^f^* ^/^ *^f^* mice were created by crossing *EpoR*‐*CreGFP* transgenic mice (Heinrich et al., 2004) (gift from U. Klingmuller and S. Orkin) with *Kcc3* ^+/^ *^f^* mice (Seja et al., 2012) (gift from T. Jentsch), then back‐crossing selected *EpoCre*; *Kcc3* ^+/^ *^f^* mice with *Kcc3* ^+/^ *^f^* or *Kcc3^f^* ^/^ *^f^* mice and selecting the desired genotype. *EpoCre*; *Kcc3^f^* ^/^ *^f^* mice were crossed next with *Kcc1* ^−/−^ or with *Kcc1* ^−/−^; *Kcc3* ^+/−^ mice (Rust et al., 2007) to yield the following intermediary genotypes:

*Kcc1* ^+/−^; *Kcc3* ^+/^ *^f^*, *Kcc1* ^+/−^; *Kcc3* ^+/^ *^f^*; *Kcc3* ^+/−^, *EpoR*‐*Cre*; *Kcc1* ^+/−^; *Kcc3* ^+/^ *^f^* and EpoR‐Cre; *Kcc1* ^+/−^; *Kcc3* ^+/^ *^f^*; *Kcc3* ^+/−^.

These intermediary genotypes were further crossbred to create the genotypes intended for physiological study:

*Kcc1* ^−/−^; *Kcc3^f^* ^/^ *^f^*, *EpoR*‐*Cre*; *Kcc1* ^−/−^; *Kcc3^f^* ^/^ *^f^*, and *EpoR*‐*Cre*; *Kcc1* ^−/−^; *Kcc3* ^−/^ *^f^*.

All mice were on the C57Bl6/J background.

Mice were screened for the presence of the Cre recombinase transgene using primers EpoR.A (5'‐GTGTGGCTGCCCCTTCTGCCA‐3', Cre forward), EpoR.B (5'‐GGCAGCCTGGGCACCTTCAC‐3', EpoR1 promoter forward) and EpoR.C (5'‐CAGGAATTCAAGCTCAACCTCA‐3', EpoR1 reverse common for both alleles), as originally described (Heinrich et al., 2004).

Mice were screened for presence of the floxed KCC3 allele using primers 00226 (5'‐GTCAGTGAGTAATCACTGTGG‐3', forward) and 00224 (5'‐GAGTATGGCTGAAATTCAAGCAC‐3', reverse), targeting sequences on either side of a loxP site inserted into *Kcc3* intron 6 (Seja et al., 2012). *EpoR*‐*Cre*; *Kcc3^f^* ^/^ *^f^* progeny mice desired for the study were detected at expected Mendelian ratios. All *EpoR*‐*Cre*; *Kcc3^f^* ^/^ *^f^* mice developed and gained weight normally through ages 6–8 weeks, at which time they were exsanguinated for study.

Wild‐type C57Bl6/J mice used in the study were bred in‐house. Some were the progeny of two C57Bl6/J parents, whereas some were the progeny of the intermediate breeding steps described above, most commonly of crosses between *EpoR*‐*Cre* mice and *Kcc3* ^+/^ *^f^* mice. This heterogeneity of WT controls is a limitation of the study.

### 2.2 Preparation of erythrocytes for flux studies

Blood was collected in heparinized syringes by cardiac puncture of Avertin‐anesthetized mice according to protocols approved by the Institutional Animal Care and Use Committee of Beth Israel Deaconess Medical Center. Heparinized blood was centrifuged at low speed in microfuge tubes and buffy coats were carefully aspirated. Cells were resuspended in ~20 volumes of choline wash solution (CWS‐Mg, in mM, 172 choline Cl, 1 MgCl_2_, 10 Tris MOPS, pH 7.40 at 4°C) in 50 ml Falcon tubes and centrifuged at 2500 rpm for 5 min at 4°C. Cells were resuspended and washed 4 more times, with the repeated aspiration of residual buffy coat. Washed cells were then suspended to 30%–50% cytocrit in the wash solution and kept at 4°C for same‐day use in flux studies. Red blood cell indices were measured with the ADVIA 120 hematology analyzer, using mouse software (Siemens Diagnostic Solutions) as previously described (Shmukler et al., 2019).

### 2.3 Measurement of K^+^ efflux

KCC activity was determined as Cl^−^‐dependent K^+^ efflux from RBC either in isotonic (basal) conditions or stimulated either by hypotonic saline or by addition to isotonic saline of staurosporine (1 µM) or urea (500 mM). All media contained, in addition, 1 mM ouabain and 10 µM bumetanide, as previously described (Shmukler et al., 2019, 2020). Freshly isolated mouse RBC were incubated in either isotonic NaCl medium (containing, in mM, 160 NaCl, 1 MgCl2, 10 glucose, 10 Tris‐MOPS, pH 7.4 at 37°C) or hypotonic NaCl media (in mM, 115 NaCl, 1 MgCl_2_, 10 glucose, 10 Tris‐MOPs, pH 7.4 at 37°C). Cl^−^‐free isotonic and hypotonic media substituted equimolar Na sulfamate for NaCl and equimolar MgNO_3_ for MgCl_2_. Samples of washed RBC were resuspended in each medium at 4°C. Triplicate aliquots were then incubated in 4 ml polystyrene tubes at 37°C in each medium. After 5 or 25 min incubation, tubes containing aliquots were immediately transferred to an ice water bath, then centrifuged at 2500 rpm for 4 min at 4°C. Supernatants were used for the measurement of K content by atomic absorption (Dunham et al., 1980). K^+^ efflux into Cl^−^‐containing and into sulfamate‐containing media was calculated from slopes of the linear regression of K content versus time. K‐Cl cotransport in a given condition was calculated as the difference between K^+^ efflux into Cl^−^‐containing medium and that into Cl^−^‐free (sulfmate‐containing medium). Hypotonicity‐stimulated K‐Cl cotransport activity was estimated by subtracting Cl^−^‐dependent K^+^ efflux into the isotonic medium from that into the hypotonic medium. KCC activity stimulated by staurosporine (1 µM) or by urea (500 mM) was determined by subtracting Cl^−^‐dependent K^+^ efflux measured in the absence of these stimulatory agents from that measured in their presence.

**FIGURE 1:**
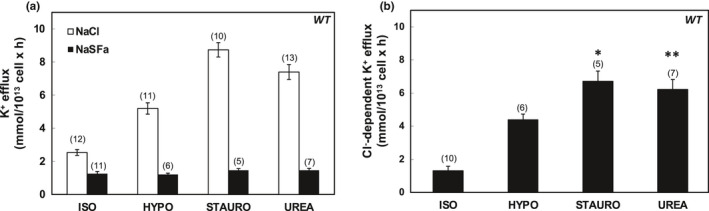
K‐Cl cotransport activity in erythrocytes of Wild Type (WT) mice. (a) K^+^ efflux measured in the presence of NaCl (open bars) and Na sulfamate (SFa; black bars) in basal isotonic conditions (ISO) and after stimulation by hypotonic media (HYPO), or by supplementation of isotonic media with 1 µM staurosporine (STAURO) or with 500 mM urea. (b) K‐Cl cotransport activity measured as Cl^−^‐dependent K^+^ efflux activity in ISO, HYPO, STAURO, and UREA conditions, calculated as the difference in K^+^ efflux values in the presence of Cl^−^ and SFa as shown in panel (a). Values are mean + SEM for (*n*) assays, each measured in triplicate. Each assay represented pooled blood from 2–3 mice. Cl^−^‐dependent K^+^ efflux values were compared by Krustal–Wallis ANOVA with Dunn's correction for multiple comparisons, yielding *p = 0.028 and **p = 0.007 verus ISO. By Mann–Whitney unpaired *t*‐test, ISO differed from HYPO (*p *< 0.002), from STAURO (*p *= 0.0007), and urea (*p *= 0.0001). Differences in values of (*n*) in panels (a) and (b) in this and subsequent figures reflect occasional experimental days on which blood volume obtained from terminally exsanguinated mice was inadequate to complete all planned experimental efflux conditions

**FIGURE 2:**
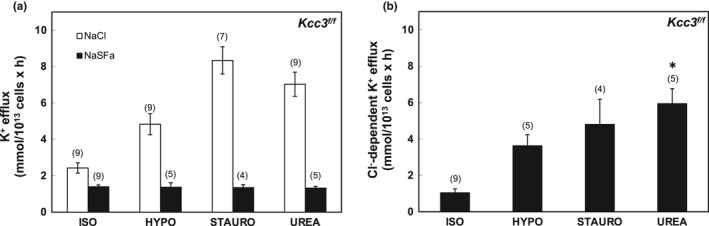
K‐Cl cotransport activity in erythrocytes of *Kcc3^f^* ^/^ *^f^* mice. (a) K^+^ efflux measured in the presence of NaCl (open bars) and Na sulfamate (SFa; black bars) in basal ISO and stimulatory HYPO, STAURO, or UREA conditions. (b) K‐Cl cotransport activity measured as Cl^−^‐dependent K^+^ efflux activity in ISO, HYPO, STAURO, and UREA conditions, calculated as the difference in K^+^ efflux values in the presence of Cl^−^ and SFa as shown in panel (a). Values are mean ± SEM for (*n*) assays, each measured in triplicate. *p < 0.001 versus ISO by Kruskal–Wallis ANOVA with Dunn's correction for multiple samples. Unpaired Mann–Whitney *t*‐tests revealed differences between ISO and HYPO (*p* < 0.002) and UREA (*p* < 0.001)

**FIGURE 3:**
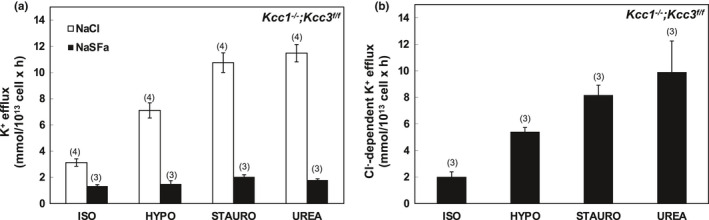
K‐Cl cotransport activity in erythrocytes of *Kcc1* ^−/−^; *Kcc3^f^* ^/^ *^f^* mice. (a) K^+^ efflux was measured in the presence of NaCl (open bars) and Na sulfamate (SFa; black bars) in basal ISO and stimulatory HYPO, STAURO, or UREA conditions. (b) K‐Cl cotransport activity measured as Cl^−^‐dependent K^+^ efflux activity in ISO, HYPO, STAURO, and UREA conditions, calculated as the difference in K^+^ efflux values in the presence of Cl^−^ and SFa as shown in panel (a). Values are mean ± SEM for (*n*) assays, each measured in triplicate. Although the magnitudes of stimulation of Cl^−^‐dependent K^+^ efflux trended toward the expected values, none of the stimulated conditions differed significantly from ISO by Kruskal–Wallis ANOVA or by unpaired Mann–Whitney *t*‐tests, (likely reflecting low “*n*”)

**FIGURE 4:**
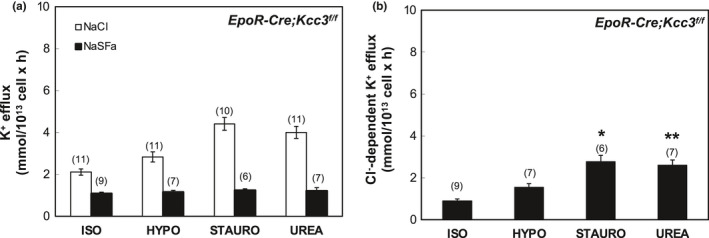
K‐Cl cotransport activity in erythrocytes of *EpoR*‐*Cre*; *Kcc3^f^* ^/^ *^f^* mice. (a) K^+^ efflux was measured in the presence of NaCl (open bars) and Na sulfamate (SFa; black bars) in basal ISO conditions and in stimulatory HYPO, STAURO) or UREA conditions. (b) K‐Cl cotransport activity measured as Cl^−^‐dependent K^+^ efflux activity in ISO, HYPO, STAURO, and UREA conditions, calculated as the difference in K^+^ efflux values in the presence of Cl^−^ and SFa as shown in panel (a). Values are mean + SEM for (*n*) assays, each measured in triplicate. *p = 0.019; **p = 0.023 versus ISO by Krusal–Wallis ANOVA with Dunn's correction. Mann–Whitney unpaired *t*‐test indicated that ISO differed from HYPO (*p* = 0.008), STAURO (*p* = 0.0004) and UREA (*p* = 0.0003)

**FIGURE 5:**
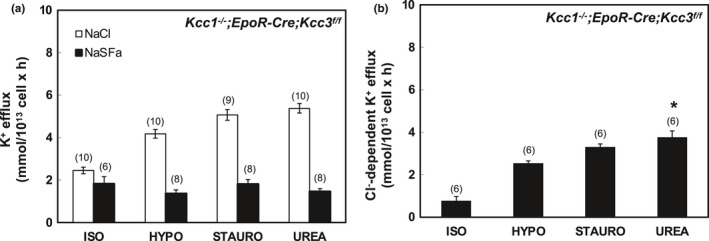
K‐Cl cotransport in erythrocytes of *Kcc1* ^−/−^; *EpoR*‐*Cre*; *Kcc3^f^* ^/^ *^f^* mice. (a) K^+^ efflux was measured in the presence of NaCl (open bars) and Na sulfamate (SFa; black bars) in basal ISO conditions and in stimulatory HYPO, STAURO) or UREA conditions. (b) K‐Cl cotransport activity measured as Cl^−^‐dependent K^+^ efflux activity in ISO, HYPO, STAURO, and UREA conditions, calculated as the difference in K^+^ efflux values in the presence of Cl^−^ and SFa as shown in panel (a). Values are mean ± SEM for (*n*) assays, each measured in triplicate. *p = 0.02 versus ISO by Kruskal–Wallis ANOVA with Dunn's correction. However, by Mann–Whitney unpaired *t*‐test ISO differed from HYPO, STAURO, and UREA (*p* = 0.002 for each)

**FIGURE 6:**
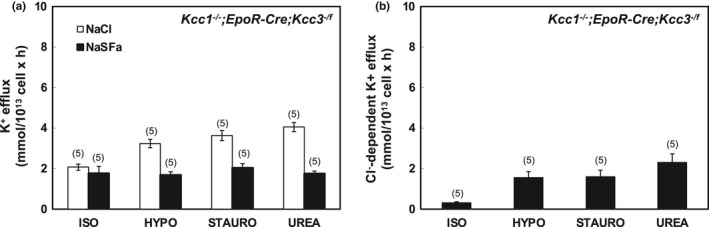
K‐Cl cotransport in erythrocytes of *Kcc1* ^−/−^; *EpoR*‐*Cre*; *Kcc3* ^−/^ *^f^* mice. (a) K^+^ efflux was measured in the presence of NaCl (open bars) and Na sulfamate (SFa; black bars) in basal ISO conditions and in stimulatory HYPO, STAURO) or UREA conditions. (b) K‐Cl cotransport activity measured as Cl^−^‐dependent K^+^ efflux activity in ISO, HYPO, STAURO, and UREA conditions, calculated as the difference in K^+^ efflux values in the presence of Cl^−^ and SFa as shown in panel (a). Values are mean ± SEM for (*n*) assays, each measured in triplicate. Although HYPO, STAURO, and UREA values were statistically indistinguishable from ISO by Kruskal–Wallis ANOVA, each stimulated condition differed from ISO by Mann–Whitney *t*‐test (*p* = 0.008 for each)

In Figures 1, 2, 3, 4, 5, 6, panels a present results measured in the presence and in the absence of chloride media (with sulfamate as substituent anion). Panels b in Figures 1, 2, 3, 4, 5, 6 present the Cl^−^‐dependent fraction of K^+^ efflux, calculated only from the smaller number of experiments in which blood volumes permitted measurements in both ionic conditions (i.e., in both chloride and sulfamate media).

### 2.4 Measurement of RBC ion content

Intracellular contents of Na and K were determined in freshly isolated RBC by atomic absorption spectrophotometry (Analyst 800; PerkinElmer) as described (Shmukler et al., 2019, 2020). RBC was washed five times in CWS‐Mg media, and an aliquot was used for manual determination of hematocrit. Lysates of RBC suspensions diluted 1:50 (for cell Na determination) and 1:500 (for cell K determination) were prepared in 0.02% Acationox, clarified by centrifugation at 3000 rpm, and stored at 4°C for later atomic absorption spectrophotometry.

### 2.5 Statistics

Data were analyzed by Kruskal–Wallis ANOVA with Dunn's correction for multiple comparisons. As “*n*” was below <30 for measurement of Cl^−^‐dependent K^+^ efflux, these data were also analyzed by Mann–Whitney non‐parametric unpaired *t*‐test.

## 3 RESULTS

### 3.1 Effects of erythroid‐specific *Kcc3* inactivation on hematological indices and ion content

*Kcc3^f^* ^/^ *^f^* mouse RBC had normal hematological indices (Table 1), whereas *EpoR*‐*Cre*; *Kcc3^f^* ^/^ *^f^* mouse RBC exhibited a slightly reduced hematocrit without change in percentage reticulocytes. The slight macrocytosis evident in *Kcc1* ^−/−^; *EpoR*‐*Cre*; *Kcc3^f^* ^/^ *^f^* mouse RBC as compared to *Kcc3^f^* ^/^ *^f^* mouse RBC was further increased in RBC of *Kcc1* ^−/−^; *EpoR*‐*Cre*; *Kcc3* ^−/^ *^f^* mice. These modest increases in MCV were reflected in slightly lower CHCM in RBC of *Kcc1* ^−/−^; *EpoR*‐*Cre*; *Kcc3* ^−/^ *^f^* mice than in RBC of *Kcc1* ^−/−^; *EpoR*‐*Cre*; *Kcc3^f^* ^/^ *^f^* mice. The few hyperchromic cells detected among WT RBC were reduced in *EpoR*‐*Cre*; *Kcc3^f^* ^/^ *^f^* RBC, and were undetectable in RBC of both *Kcc1* ^−/−^; *EpoR*‐*Cre*; *Kcc3^f^* ^/^ *^f^* mice and *Kcc1* ^−/−^; *EpoR*‐*Cre*; *Kcc3* ^−/^ *^f^* mice (Table 1). These changes were consistent with those previously observed in RBC of *Kcc3* ^−/−^ mice and of *Kcc1* ^−/−^; *Kcc3* ^−/−^ mice (Rust et al., 2007; Shmukler et al., 2019).

**TABLE 1:** ADVIA120^TM^ erythrocyte parameters in different mouse genotypes

Genotype	Hcrit (%)	MCV (fl)	CHCM (g/dl)	RDW (%)	Hyperchromic (%)	Retics (%)
WT (9)	41.4 ± 0.8	49.9 ± 0.6	28.4 ± 0.2	13.1 ± 0.4	0.2 ± 0.1	3.6 ± 0.3
*KCC3^f^* ^/^ *^fl^* (25)	43.2 ± 0.5	49.7 ± 0.5	28.2 ± 0.2	13.8 ± 0.2	0.1 ± 0.0	3.0 ± 0.2
*EpoR‐Cre KCC3^f^* ^/^ *^f^* (29)	39.7 ± 0.5^ƒƒ^	49.6 ± 0.5	27.8 ± 0.1	13.1 ± 0.2	0.1 ± 0.0*	3.0 ± 0.1
*KCC1* ^−/−^; *KCC3^f^* ^/^ *^f^* (7)	44.7 ± 1.3^†^	49.3 ± 1.3	28.3 ± 0.2	13.8 ± 0.5	0.1 ± 0.0	2.8 ± 0.2
*KCC1* ^−/−^; *EpoR‐Cre; KCC3^f^* ^/^ *^f^* (21)	41.9 ± 0.4	53.0 ± 0.5^ƒƒ,††^	27.7 ± 0.2	12.8 ± 0.3^ƒ^	0.0 ± 0.0**	2.7 ± 0.2
*KCC1* ^−/−^; *EpoR‐Cre; Kcc3* ^−/^ *^f^* (10)	43.0 ± 0.6^†^	55.8 ± 0.7**^,ƒƒ, ††, ¶¶^	27.2 ± 0.2**^,†,¶^	12.8 ± 0.2	0.0 ± 0.0**	2.9 ± 0.3

Compared to WT (*p < 0.04, **p < 0.007).

Compared to KCC3^f/f^ (^ƒ^ *p* < 0.004, ^ƒƒ^ *p* < 0.0003).

Compared to EpoRCre; KCC3^f/f^ (†*p *< 0.005, ††*p *< 0.0007).

Compared to Kcc1^‐/‐^; Kcc3^f/f^ (¶*p *< 0.03, ¶¶*p *< 0.001).

ANOVA: non‐parametric Kruskal–Wallis test with Dunn's correction for multi‐comparison test.

Atomic absorption spectrometric measurement of ion content (Table 2) revealed in RBC of *Kcc1* ^−/−^; *EpoR*‐*Cre*; *Kcc3^f^* ^/^ *^f^* mice and *Kcc1* ^−/−^; *EpoR*‐*Cre*; *Kcc3* ^−/^ *^f^* mice only modest, apparent increases in K content that failed to achieve statistical significance, consistent with our earlier observations suggesting compensatory changes in K‐Cl cotransport (Rust et al., 2007; Shmukler et al., 2019) or related activities. RBC of *Kcc1* ^−/−^; *Kcc3^f^* ^/^ *^f^* mice exhibited modestly reduced Na content.

**TABLE 2:** Red cell ion content in different genotypes

Genotype	[Na^+^]_i_	[K^+^]_i_	[Mg^2+^]_i_
	(mmol/Kg Hb)		
WT (5)	19.8 ± 1.0	404.8 ± 13.3	10.3 ± 1.2
*KCC3^f^* ^/^ *^f^* (9)	19.8 ± 0.7	409.3 ± 6.4	8.9 ± 0.3
*EpoR*‐*Cre; KCC3^f^* ^/^ *^f^* (11)	20.0 ± 1	399.3 ± 14	8.5 ± 0.3
*KCC1* ^−/−^; *KCC3^f^* ^/^ *^f^* (6)	14.3 ± 1.5^†^	410.3 ± 14	7.6 ± 0.3
*KCC1* ^−/−^; *EpoR‐Cre; KCC3^fl^* ^/^ *^f^* (10)	18.1 ± 0.7	431.8 ± 10.5	8.6 ± 0.4
*KCC1* ^−/−^; *EpoR‐Cre; Kcc3* ^−/^ *^f^* (5)	22.2 ± 2.3	419.6 ± 12.4	8.6 ± 0.5

Compared to EpoRCre; KCC3^f/f^ (†*p *< 0.04).

ANOVA: non‐parametric Kruskal–Wallis test with Dunn's correction for multi‐comparison test.

### 3.2 Effects of erythroid‐specific *Kcc3* inactivation on RBC K‐Cl cotransport activity

**FIGURE 7:**
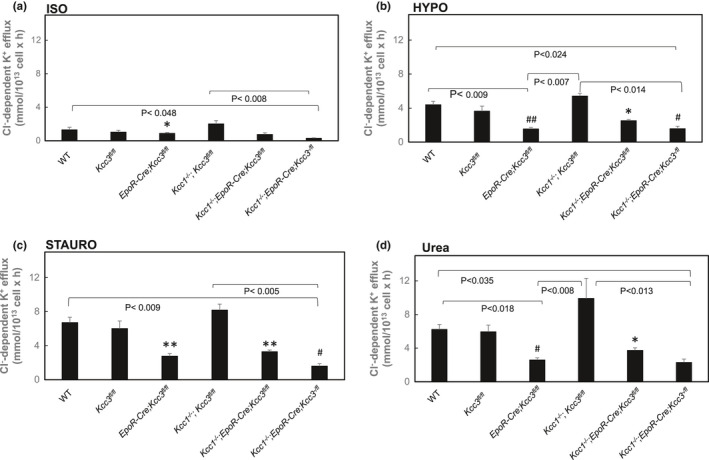
K‐Cl cotransport rates compared among mouse erythrocytes of different genotypes for each efflux condition. (a) Basal (isotonic) Cl^−^‐dependent K^+^ efflux among genotypes. (b) Hypotonicity‐stimulated Cl^−^‐dependent K^+^ efflux among genotypes. (c) Staurosporine‐stimulated Cl^−^‐dependent K^+^ efflux among genotypes. (d) Urea‐stimulated Cl^−^‐dependent K^+^ efflux among genotypes. Data are replotted from panels (b) of Figures 1, 2, 3, 4, 5, 6, with the same (*n*). *p*‐values shown for the bracketed pairwise comparisons are from Kruskal–Wallis ANOVA with Dunn's correction. Symbols indicate unpaired Mann–Whitney *t*‐tests comparing genotypes within each experimental condition. *p < 0.005 versus WT; **p<0.0005 versus WT; ^#^ *p *< 0.05 versus *Kcc1* ^−/−^; *EpoR*‐*Cre*; *Kcc3^f^* ^/^ *^f^*; ^##^ *p *= 0.005 versus *Kcc1* ^−/−^; *EpoR*‐*Cre*; *Kcc3^f^* ^/^ *^f^*

As shown in Figure 1, wild‐type (WT) mouse RBC exhibited ~1 mmol/L cell × h of K‐Cl cotransport at baseline isotonic conditions. Hypotonic conditions increased isotonic K‐Cl cotransport activity >3‐fold, whereas K‐Cl cotransport activity was stimulated ~5–7‐fold by the nonspecific serine‐threonine‐tyrosine kinase inhibitor, staurosporine (1 µM), and ~4–6‐fold by the renal medullary osmolyte, urea (500 mM) (Figure 1). These fold‐stimulation values were statistically indistinguishable (Figure 7) from those observed in *Kcc3^f^* ^/^ *^f^* mouse RBC (Figure 2) and in RBC of *Kcc1* ^−/−^; *Kcc3^f^* ^/^ *^f^* mice (Figure 3). The data were consistent with lack of effect of the intronic flox site insertions into the *Kcc3* gene (Seja et al., 2012), and with our previously reported lack of effect of global *Kcc1* knockout on RBC K‐Cl cotransport activity (Rust et al., 2007; Shmukler et al., 2019).

**FIGURE 8:**
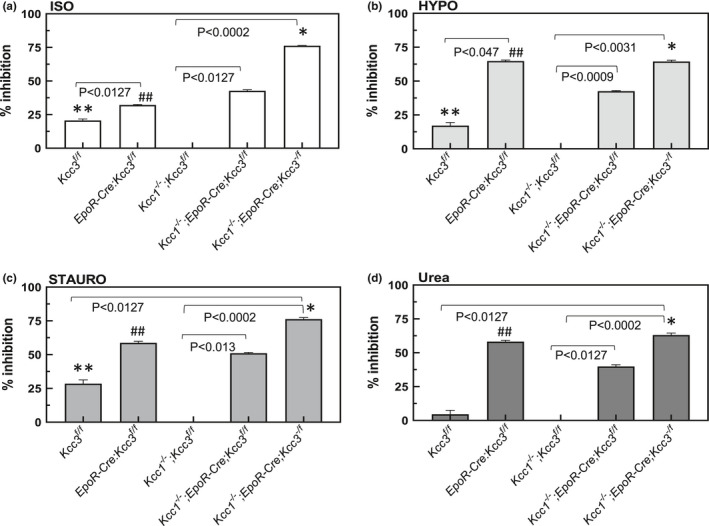
Data from Figure 7 expressed as % inhibition versus K‐Cl cotransport in WT RBC tested in the same basal (Isotonic, a) or stimulated conditions HYPO (b), STAURO (c), or UREA (d). SEM values were <1% for ISO and HYPO, <2.5% for STAURO and UREA (data not shown). *p*‐values for comparisons linked by brackets are by Kruskal–Wallis ANOVA with Dunn's correction for all genotypes within each experimental condition. Symbols indicate unpaired Mann–Whitney *t*‐tests comparing genotypes within each condition. *p = 0.008 versus *Kcc1* ^−/−^; *EpoR*‐*Cre*; *Kcc3^f^* ^/^ *^f^*; **p = 0.008 versus *Kcc1* ^−/−^; *Kcc3^f^* ^/^ *^f^*; ^##^ *p *< 0.008 versus *Kcc1* ^−/−^; *EpoR*‐*Cre*; *Kcc3^f^* ^/^ *^f^*

We next tested the effect of nominally erythroid‐specific Cre‐mediated *Kcc3* inactivation on RBC K‐Cl cotransport activity. RBC of *EpoR*‐*Cre*; *Kcc3^f^* ^/^ *^f^* mice exhibited ~35% reduction in basal (isotonic) K‐Cl cotransport (Figure 4) as compared to WT levels (Figure 1). Hypotonicity‐stimulated K‐Cl cotransport was reduced by 65% as compared to that in WT RBC (Figures 4, 7 and 8). Staurosporine‐stimulated K‐Cl cotransport and urea‐stimulated K‐Cl cotransport in *EpoR*‐*Cre*; *Kcc3^f^* ^/^ *^f^* RBC were each reduced 58% as compared to those activities in WT RBC (Figures 4, 7 and 8).

If penetrance of erythroid‐specific Kcc3 inactivation based on Cre‐mediated excision is complete, then the reduction in erythroid K‐Cl cotransport activity should be equivalent to that achieved by global knockout. Figure 5 presents K‐Cl cotransport measured in RBC of *Kcc1* ^−/−^; *EpoR*‐*Cre*; *Kcc3^f^* ^/^ *^f^* mice. Figure 6 presents K‐Cl cotransport measured in RBC of *Kcc1* ^−/−^; *EpoR*‐*Cre*; *Kcc3* ^−/^ *^f^* mice. Comparison of Figures 5 and 6 reveals reduction of RBC K‐Cl cotransport to a considerably greater degree in *Kcc1* ^−/−^; *EpoR*‐*Cre*; *Kcc3* ^−/^ *^f^* mice than in *Kcc1* ^−/−^; *EpoR*‐*Cre*; *Kcc3^f^* ^/^ *^f^* mice. Thus, in the absence of functional KCC1, the presence of two floxed *Kcc3* alleles reduced basal (unstimulated) K‐Cl cotransport by 43%, whereas in the presence of one floxed *Kcc3* allele and one null *Kcc3* allele, basal K‐Cl cotransport was reduced by 76% (Figures 7 and 8).

The functional difference between EPOR‐Cre‐mediated targeted excision of *Kcc3* and germline global knockout of *Kcc3* is further supported by the examination of differential reductions of stimulated KCC3 function. Thus, comparison of stimulated K‐Cl cotransport in RBC of *Kcc1* ^−/−^; *EpoR*‐*Cre*; *Kcc3^f^* ^/^ *^f^* mice with that in RBC of *Kcc1* ^−/−^; *EpoR*‐*Cre*; *Kcc3* ^−/^ *^f^* mice reveal respective reductions in hypotonic stimulation of K‐Cl cotransport by 43% and 64% as compared to values in WT RBC. Similar comparisons of staurosporine‐stimulated K‐Cl cotransport in RBC of *Kcc1* ^−/−^; *EpoR*‐*Cre*; *Kcc3^f^* ^/^ *^f^* mice and in RBC of *Kcc1* ^−/−^; *EpoR*‐*Cre*; *Kcc3* ^−/^ *^f^* mice revealed respective reductions of 51% and 76% versus values in WT RBC. Respective reductions in urea‐stimulated K‐Cl cotransport were 40% and 63% versus values in WT RBC (Figures 7 and 8). These data concur in suggesting incomplete penetrance of EPOR‐Cre‐mediated excision of the floxed *Kcc3* gene in the erythroid lineage of these mice, as measured by basal and stimulated K‐Cl cotransport in mature circulating RBC.

We previously observed that RBC of *Kcc3* ^−/−^ global knockout mice exhibited loss of more than half of measurable K‐Cl cotransport. The additional global genetic inactivation of *Kcc1* in *Kcc3* ^−/−^ global knockout mice completely suppressed erythroid K‐Cl cotransport activity (Rust et al., 2007). In the current study, when EpoR‐Cre‐mediated homozygous inactivation of *Kcc3* was accompanied by global inactivation of *Kcc1*, hypotonic stimulation of K‐Cl cotransport was reduced from 65% in RBC of *EpoR*‐*Cre*; *Kcc3^f^* ^/^ *^f^* mice to 43% in RBC of *Kcc1* ^−/−^; *EpoR*‐*Cre*; *Kcc3^f^* ^/^ *^f^* mice (values with respect to WT RBC K‐Cl cotransport). Inhibition of urea‐stimulated WT K‐Cl cotransport was similarly reduced from 58% to 40% of WT values. Inhibition of staurosporine‐stimulated WT K‐Cl cotransport was less remarkably reduced from 59% to 51% of WT values (Figures 7 and 8).

We note that although global *Kcc1* inactivation in *Kcc3^f^* ^/^ *^f^* mice “prevented” the small reduction in K‐Cl cotransport noted in RBC of *Kcc3^f^* ^/^ *^f^* mice (Figures 7 and 8; see also Figures 2 and 3), this small reduction did not achieve statistical significance.

## 4 DISCUSSION

We have cross‐bred mice to generate erythroid‐specific genetic inactivation of the gene encoding the SLC12 K‐Cl cotransporter, KCC3/SLC12A6. To our knowledge, this report represents the first use of the Cre recombinase under the control of the promoter of the gene encoding the erythropoietin receptor (EPOR) to inactivate a gene encoding a polytopic membrane protein, solute transporter, or ion channel. The *EpoR*‐*Cre*; *Kcc3^f^* ^/^ *^f^* mouse exhibited a mild reduction in hematocrit without significant reticulocytosis, with little difference from the erythrocyte indices observed in RBC of the *Kcc3* ^−/−^ global knockout mouse. RBC K content was not significantly increased by erythroid‐specific inactivation of *Kcc3*, consistent with our previous observations suggestive of possible functional compensation by KCC1/SLC12A4 (Rust et al., 2007; Shmukler et al., 2019).

Although basal isotonic K^+^ efflux in *Kcc3^f^* ^/^ *^f^* RBC appeared at first glance similar to that in WT RBC (Figures 1 and 2), assessment of K‐Cl cotransport as Cl^−^‐dependent K^+^ efflux revealed ~20% reduction of basal K‐Cl cotransport in Kcc3^f/f^ RBC (Figures 7 and 8). As previously described in Kcc1^−/−^ mouse RBC (Rust et al., 2007), global inactivation of KCC1 in Kcc1^−/−^; Kcc3^f/f^ mice led to no further decrease in K‐Cl cotransport of RBC of Kcc3^f/f^ mice (Figures 2 and 3). In contrast, K‐Cl cotransport in RBC from *EpoR*‐*Cre*; *Kcc3^f^* ^/^ *^f^* mice, in which KCC3 was inactivated only in the erythroid lineage, was reduced ~65% below levels of K‐Cl cotransport in RBC with intact KCC3 (Figures 2 and 4). This value corresponds well to the reduction in K‐Cl cotransport previously reported in *Kcc3* ^−/−^ (global knockout mouse) RBC. However, unlike erythroid K‐Cl cotransport in *Kcc3* ^−/−^ mice (Rust et al., 2007), in which global inactivation of KCC1 led to complete loss of RBC K‐Cl cotransport, stimulated K‐Cl cotransport in RBC of *Kcc1* ^−/−^; *EpoR*‐*Cre*; *Kcc3^f^* ^/^ *^f^* mice was no lower in magnitude than in RBC of EpoR‐Cre; Kcc3^f/f^ mice (Figure 5) and, indeed, revealed increased K‐Cl cotransport in response to some stimuli (Figures 7 and 8). The cause of this paradoxical effect of KCC1 genetic inactivation remains unclear. KCC1 knockout was previously reported to increase KCC3B polypeptide accumulation (Rust et al., 2007). In the current setting of EpoR‐Cre‐mediated *Kcc3* inactivation, this mechanism is not plausible if EpoR‐Cre‐mediated recombination is fully penetrant.

Indeed, while EpoR‐Cre‐mediated recombination exhibited near 94% recombination efficiency in fetal liver erythroblasts, recombination efficiency was only 62% in adult spleen erythroblasts and 76% in adult bone marrow erythroblasts (Heinrich et al., 2004). The latter recombination efficiencies might apply better to the adolescent and adult mice from which blood was sampled for measurement of K‐Cl cotransport. A later report documented even lower recombination efficiencies in early and, especially, late erythroblasts (Usenko et al., 2014). We, therefore, tested the functional equivalence in RBC of germ‐line and erythroid‐specific knockout of *Kcc3*. As shown in Figures 5 and 6, and summarized in Figures 7 and 8, K‐Cl cotransport in RBC of *Kcc1* ^−/−^; *EpoR*‐*Cre*; *Kcc3^f^* ^/^ *^f^* mice was greater than that measured in RBC of *Kcc1* ^−/−^; *EpoR*‐*Cre*; *Kcc3* ^−/^ *^f^* mice.

This result strongly suggests that EpoR‐Cre mediated inactivation of erythroid Kcc3 was incomplete in our mouse colony. A similar comparison of *f*/*f* versus *f*/Δ genotypes has been used to document ~70% Cre excision efficiency of iCre driven by the male germ cell‐specific *Stra8* promoter (Usenko et al., 2014). Moreover, a smooth muscle‐specific SM22 α‐driven tamoxifen‐inducible Cre has been noted variably to leave target protein levels unchanged despite documented appropriate excision efficiency (Turlo et al., 2010). Alternatively, the presence and/or activity of Cre may have led to upregulatory compensation of K‐Cl activity in the *f*/*f* mice. This higher‐than‐expected K‐Cl activity might represent upregulation of KCC4 expression, normally absent from mouse RBC (Rust et al., 2007) but present in human RBC (Pan et al., 2011). Thus, future work should compare the erythroid expression of plasmalemmal KCC3, KCC1, and KCC4 in wild‐type, *Kcc3* ^−/−^, and *f*/*f* mice. Alternatively, the elevated K‐Cl cotransport might reflect an altered balance between activities of the WNK/SPAK/OSR1 kinase pathway that inhibits K‐Cl cotransporters and the incompletely defined serine‐threonine phosphatases that stimulate K‐Cl cotransporters, together tightly regulating both K‐Cl and Na‐K‐Cl cotransport in red cells, and numerous additional transporters and channels throughout the body (Alessi et al., 2014; Frenette‐Cotton et al., 2018; Los et al., 2018).

Although not reported in the context of previous use of EpoR‐Cre to study hematopoiesis, we cannot exclude the possibility that Cre expression during mid‐late hematopoiesis might alter K‐Cl cotransport expression or activity. Cre toxicity observed in cell culture led to early strategies for self‐excision to avoid prolonged expression (Silver & Livingston, 2001). Apparently, idiosyncratic Cre toxicity has indeed been observed in early hematopoietic cells in the context of some, but not all, promoters (Naiche & Papaioannou, 2007). Cre toxicity in cardiomyocytes (Pugach et al., 2015) and other tissues (Balkawade et al., 2019; Zappia et al., 2016) can be attenuated by endogenous factors (Hall et al., 2012; Hull et al., 2013) and exacerbated by drugs commonly used for temporal control of gene excision (Benedykcinska et al., 2016). Furthermore, Cre‐mediated recombination can be influenced by target locus, the distance between and sequences flanking LoxP sites, level of Cre activity in individual cells, and parental sex of the Cre donor animal (for which we did not control) (Liu et al., 2013; Luo et al., 2020). We attribute the apparently reduced Cre‐mediated recombination frequency in erythroid precursors in our current work to some combination of Cre itself and its expression and/or function in those erythroid precursors, rather than to characteristics of the floxed *Kcc3* allele, which has previously demonstrated successful Cre‐mediated recombination in brain (Seja et al., 2012).

As *EpoR*‐*Cre*; *Kcc3^f^* ^/^ *^f^* mice are grossly normal and appear normally fertile, we achieved our initial study objective in creating an erythroid‐specific knockout of *Kcc3*, the investigation of which would not be impaired by the central and peripheral nervous system consequences of global *Kcc3* knockout. This achievement allows consideration of the use of EpoR‐Cre to generate highly erythroid‐specific knockout of other RBC membrane proteins of interest. However, the apparent variation of EpoR‐Cre recombination efficiency dependent on stages of embryonic and erythropoietic development, perhaps reflecting shifts in tissue site predominance of erythropoiesis, may counsel caution in the choice of promoters for erythroid‐specific transcription of Cre recombinase in the generation of erythroid‐specific knockout mice to be studied as adults.

## CONFLICTS OF INTEREST

Seth L. Alper was supported by NIH grant HL077765 and research funds from Quest Diagnostics. Jay G. Wohlgemuth and Jeffrey S. Dlott are employees and stockholders of Quest Diagnostics. L. Michael Snyder and Seth L. Alper are consultants to Quest Diagnostics.

## ETHICAL STATEMENT

All mouse studies reported were performed according to protocols approved by the Institutional Animal Care and Use Committee of Beth Israel Deaconess Medical Center.

## AUTHOR CONTRIBUTIONS

BES, AR, KN, and AH performed experiments. SLA, BES, and CB conceived and designed the project. BES and AR analyzed experimental results. SLA, AR, and BES wrote the manuscript. SLA, JGW, JSD, LMS, and CB critiqued and revised the manuscript.
